# Supplementary material for: Patterns of Intron Gain and Loss in Fungi
Source: PLoS Biol. 2004 Nov 30;2(12):e422. doi: 10.1371/journal.pbio.0020422 (PMC532390; doi:10.1371/journal.pbio.0020422)
Supplement: Table S1 — Also available at http://genes.mit.edu/NielsenEtAl/. (4.3 MB ZIP). [file pbio.0020422.st001.zip › NielsenEtAl/html/451.html]

AN1757.1.NCU05942.1.MG08343.1.FG05365.1


```
 CLUSTAL W (1.82) Multiple Sequence Alignments - Introns Inserted


Sequence 1: NCU05942.1	251 aa
Sequence 2: MG08343.1	251 aa
Sequence 3: FG05365.1	251 aa
Sequence 4: AN1757.1	255 aa
Alignment Length: 255 aa
Number Identitical Residues: 218 aa
Alignment Score (without introns) 8528


MG08343.1 	MSRRYDSR0TTIFSPEGRLYQVEYALEAISHAGTAIGILAKDGIVLAAERKVTSKLLEQD
NCU05942.1	MARRYDSR0TTIFSPEGRLYQVEYALEAISHAGTAIGILAKDGIVLAAERKVTSKLLEQD
FG05365.1 	MSRRYDSR0TTIFSPEGRLYQVEYALEAISHAGTAIGILAKDGIVLAAERKVTSKLLEQD
AN1757.1  	MSRRYDSR0TTIFSPEGRLYQVEYALEAISHAGTALGILAKDGIVLAAEKKVTSKLLEQD
          	*:****** **************************:*************:**********

MG08343.1 	TSAEKLYILND~NMICAVAGMTADANILINFARQNAQRYLLTYNEDIPCEQLVRRLCDLK
NCU05942.1	TSAEKLYILND2NMICAVAGMTADANILINYARQAAQRYLLTYNEDIPCEQLVRRLCDLK
FG05365.1 	TSAEKLYILND2NMICAVAGMTADANILINYARQAAQRYLLTYNEDIPCEQLVRRLCDLK
AN1757.1  	TSAEKLYTLND2NMICAVAGMTADANILINYARQAAQRYLITYGEEIPCEQLVRRLCDLK
          	******* *** ******************:*** *****:**.*:**************

MG08343.1 	QGYTQHGGLRPFGVSFIYAGWDPQRQFQLYLSNPSGNYGGWKATSVGANNASAQSLLKQD
NCU05942.1	QGYTQHGGLRPFGVSFIYAGWDPQRQFQLYLSNPSGNYGGWKATSAGANNASAQSLLKQD
FG05365.1 	QGYTQHGGLRPFGVSFIYAGWDPRRQFQLYLSNPSGNYGGWKATSAGANNASAQSLLKQD
AN1757.1  	QGYTQHGGLRPFGVSFIYAGYDHLREFQLYQSNPSGNYGGWKATSVGANNASAQSLLKQD
          	********************:*  *:**** **************.**************

MG08343.1 	YKEDCSLKEACGMAVKVLSKTMDSTKLSSEKI1EFATVGQTKDGKVYHRLWSADEITALL
NCU05942.1	YKEDCTLKEACAMAVKVLSKTMDSTKLSPEKI1EFATVGQTKDGKIYHRLWSADEITALL
FG05365.1 	YKEDCTLKEACGMAVKVLSKTMDSTKLSSEKI1EFATVGQTEDGKIYHRLWSADEITALL
AN1757.1  	YKEDCDLKEACAMAVKVLSKTMDSTKLSSEKI1EFATVGKTKEGKIYHHLWNADEINALL
          	***** *****.****************.*** ******:*::**:**:**.****.***

MG08343.1 	KEHDLAKDDSTDDK----
NCU05942.1	KEHDLAKDDNTEDK----
FG05365.1 	KEHDLAKNEETEEK----
AN1757.1  	KEHGLAKVDDEPEAGDIK
          	***.*** :.  : .. .
```
